# Gastric Cancer Profile in Kashmiri Population With Special Dietary Habits

**DOI:** 10.1155/DTE.6.83

**Published:** 2000

**Authors:** G. M. Malik, M. Mubarik, S. A. Kadla, H. A. Durrani

**Affiliations:** 1 Department of Medicine Govt. Medical College Srinagar Kashmir 190010 India; 2 C/o Nalband Pora Safa Kadal Srinagar Kashmir 190010 India

## Abstract

The present study is a comprehensive retrospective analysis of 1341 gastric neoplasms out of 10 733 individuals subjected to upper gastrointestinal endoscopy at the main teaching cum referral hospital in the Kashmir Valley. Of these 78% were males and 22% females, majority being in the age group of 41–60 years with 60% of the patients being smokers. On endoscopy, the commonest site of cancer was the body of stomach 40.7%, followed by the antrum 35.5% and the cardiac region 23.8%. Endoscopic features revealed nodular masses 39%, polypoid masses 21%, malignant ulcers 11%, infiltrative masses 12%, rounded tumor masses 9%, linitus plastica 5% and early gastric carcinoma 3%. Histology revealed adenocarcinoma 91%, (including mucoid carcinoma 9%, and schirrous carcinoma 7%), leiomyosarcoma 7%, and reticulum cell sarcoma 2%. No significant association between *Helicobacter pylori* infection and gastric cancer was observed in a short study out of these patients. The peculiar geography and some special dietary habits with a possible familial predisposition may have a bearing on the high risk of gastric cancer in the valley.


*Diagnostic and Therapeutic Endoscopy*, Vol. 6, pp. 83–86
Reprints available directly from the publisher
Photocopying permitted by license only

© 2000 OPA (Overseas Publishers Association) N.V.
Published by license under
the Harwood Academic Publishers imprint,
part of The Gordon and Breach Publishing Group.
Printed in Malaysia.

### Gastric Cancer Profile in Kashmiri Population with Special Dietary Habits

G.M. MALIK, M. MUBARIK*, S.A. KADLA and H.A. DURRANI

*Department of Medicine, Govt. Medical College, Srinagar – 190010, Kashmir, India*

*(Received 25 February 1999; Revised 8 June 1999; In final form 12 August 1999)*

**The present study is a comprehensive retrospective analysis of 1341 gastric neoplasms out of 10 733 individuals subjected to upper gastrointestinal endoscopy at the main teaching cum referral hospital in the Kashmir Valley. Of these 78% were males and 22% females, majority being in the age group of 41–60 years with 60% of the patients being smokers. On endoscopy, the commonest site of cancer was the body of stomach 40.7%, followed by the antrum 35.5% and the cardiac region 23.8%. Endoscopic features revealed nodular masses 39%, polypoid masses 21%, malignant ulcers 11%, infiltrative masses 12%, rounded tumor masses 9%, linitus plastica 5% and early gastric carcinoma 3%. Histology revealed adenocarcinoma 91%, (including mucoid carcinoma 9%, and schirrous carcinoma 7%), leiomyosarcoma 7%, and reticulum cell sarcoma 2%. No significant association between *Helicobacter pylori* infection and gastric cancer was observed in a short study out of these patients. The peculiar geography and some special dietary habits with a possible familial predisposition may have a bearing on the high risk of gastric cancer in the valley.**

*Keywords:* Cancer, Dietary, Endoscopy, *Helicobacter pylori*, Histology, Gastric

### INTRODUCTION

The incidence of gastric cancer in the United States has decreased considerably over the past 60 years [1,2]. However, the incidence rate is comparatively high in Japan, China, Chile and Ireland [1,3]. Migrants from high to low incidence countries show a significant decrease in gastric cancer occurence in their off-springs, suggesting that the cause is related to environmental factor starting early in life [1,4]. The clinical experience has revealed a very high prevalence of gastric cancer in Kashmir, but there is scarcity of data available at present in this regard. This study describes our experience with gastric neoplasms at the main teaching cum referral hospital in Kashmir, with the inhabitants having special personal and dietary habits.

### MATERIALS AND METHODS

The study comprised 1341 patients of gastric cancer, diagnosed out of 10 733 individuals subjected to

* Corresponding author. C/o Nalband Pora, Safa Kadal, Srinagar – 190002, Kashmir, India. Tel.: +91-194-474235.

upper gastrointestinal (GI) endoscopy over the past 10 years at SMHS Hospital, Srinagar. Patients whose clinical presentation warranted a diagnosis or exclusion of gastric cancer were selected for the study. Symptoms such as anorexia, weight loss of recent onset, presistent abdominal pain, post-prandial abdominal distension, dysphagia and history of upper GI bleed were considered suggestive of a possible gastric neoplasm. The clinical examination included degree of cachexia and anemia, cervical lymphadenopathy, abdominal mass, signs of gastric outlet obstruction, hepatomegaly or ascites. The investigations included hemogram, stool examination for occult blood, upper GI radiography (in only 754 patients) and upper GI endoscopy with endoscopic biopsy for histological study. In a short study of 50 gastric cancer patients, the biopsy specimens were also studied for *Helicobacter pylori* infection and compared with 30 age/sex matched controls. The specimens for detecting *H. pylori* infection were subjected to three different test procedures including one minute endoscopy room test (OMERT), bacteriology for gram staining with histology being the gold standard [5].

TABLE I Clinical spectrum, endoscopic features and histology in gastric cancer patients

| Parameter | % of patients |
|---|---|
| *Symptomatology* | |
| Dyspepsia–anorexia | 80 |
| Weight loss | 69 |
| Upper GI bleed | 29 |
| Dysphagia | 25 |
| Abdominal mass | 20 |
| Stasis obstruction | 0.8 |
| *Physical signs* | |
| Anemia | 80 |
| Emaciation | 60 |
| Ascites | 30 |
| Palpable mass | 20 |
| Edema feet | 12 |
| Clubbing | 0.4 |
| *Site (on endoscopy)* | |
| Body of stomach | 40.7 |
| Antrum | 35.5 |
| Cardic region | 23.8 |
| *Morphology (on endoscopy)* | |
| Nodular mass | 39 |
| Polypoid mass | 21 |
| Infiltrative lesion | 12 |
| Malignant ulcer | 11 |
| Rounded tumor mass | 9 |
| Linitus plastica | 5 |
| Early gastric carcinoma | 3 |
| *Histological typing* | |
| Adenocarcinoma (including Mucoid carcinoma 9% and Schirrous carcinoma 7%) | 91 |
| Leiomyosarcoma | 7 |
| Reticulum cell sarcoma | 2 |

### RESULTS

Majority of patients belonged to the age group of 41–60 years. Nine patients were under the age of 20 years, the youngest being a 12-year-old boy. In the entire series, 78% were males and 22% females; 60% were smokers and 40% non-smokers.

Symptomatology included anorexia, dyspepsia and weight loss in majority of patients, whereas stasis obstruction was the least common symptom. The examination revealed anemia and emaciation as major physical findings, whereas clubbing was observed to be the least common finding (Table I).

Peripheral blood film revealed hypochromic microcytic anemia. Radiology showed (1) filling defect with a mass lesion (2) an ulcer with radiological features of malignancy, presence of meniscus with irregular blunting of mucosal folds around it and absence of such classical signs of benignity such as Hampton's line, ulcer collar, mound or halo (3) rigidity, mucosal destruction and decreased distensibility of a segment of the stomach indicating an infiltrative process.

On endoscopy the commonest site of cancer was the body of stomach (40.7%) followed by the antrum (35.5%) and the cardiac region (23.8%). Morphology revealed nodular masses as the commonest (39%) and early gastric cancer as the least common (3%) finding on endoscopy. Histology revealed adenocarcinoma as the commonest type (91%) and the least common being reticulum cell sarcoma 2% (Table I). In a short study of 50

TABLE II Relationship between *H. pylori* and carcinoma stomach

| Group inference | No. of cases | *H. pylori* positivity No. (%) | Statistics |
|---|---|---|---|
| Patients | 50 | 17 (34.00) | $\chi^2 df_1 = 0.003$ |
| Controls | 30 | 10 (33.33) | $P > 0.90$ (insignificant) |

*Note*: Histology was taken as gold standard.

patients, *H. pylori* infection was observed in 17 (34%) as compared to 10 (33.33%) out of 30 matched controls, which was statistically insignificant (Table II).

### DISCUSSION

The gastric cancer formed 3.4% of all admissions to the medical and surgical wards in the main teaching cum referral hospital of the valley which seems to be very high. An earlier report has also revealed an unprecedented high incidence of gastric cancer in Kashmir [6]. The commonest sites of cancer in order of frequency were observed to be the body of stomach, the antrum and the cardiac region respectively with the commonest histological type being adenocarcinoma (91%), Khuroo *et al.* have observed the antrum to be the commonest site followed by the body and the cardiac region with adenocarcinoma completely dominating the histology (99.38%) [6]. Early gastric cancer as classified by Japan Gastroenterology society [7] was detected only in 41 patients. The unfortunate handicap in picking up early cancer was the late reporting of our patients, living in a population group, majority of whom are ignorant of even general health problems. As already reported [8], no significant association between *H. pylori* infection and gastric cancer was observed in a short study out of these patients, in confirmity with some other authors [9].

The various factors can be linked to the high risk of gastric cancer in Kashmir. First, the peculiar geography of the valley (situated at an altitude of 1800–2400 m above the sea level) [10], with severe cold winter may have a bearing on the etiology. Further, the intake of a large quantity of rice by a Kashmiri native may lead to delayed gastric emptying, which can also be accounted for a possible predisposition. A few instances of familial predisposition points towards a genetic background. But, the most important of the predisposing factors may be the special dietary habits of the Kashmiris. Some earlier studies have also strengthened the view that food habits and lifestyle are closely associated with a high incidence of oesophagogastric cancer [6,11]. The excessive intake of hot salted alkaline tea, consumption of a dried leafy vegetable – *Brassica olerecea* (Haak), pickled vegetables, dried smoked fish, dried raw food, spice cakes (Wur), use of red chillies and spices are some of the distinctive dietary habits. Common salt (NaCl) is a well known irritant of gastric epithelium and has been considered to be risk factor for gastric cancer [12]. Further, N-nitroso compounds have been found in significant quantity in the dietary items like "Haak", dried raw foods, red chillies and "Wur" [13,14], consumed by the Kashmiris. In addition salt tea has been found to contain high amounts of N-nitrosopipecolic acid and other unidentified non-volatile N-nitrosocompounds. The presence of N-nitroso compounds (found in most of the customary dietary items of a native Kashmiri) in the stomach has been incriminated as a possible etiological factor in the genesis of gastric cancer [15,16].

In conclusion, we noticed that gastric cancer is a frequently observed neoplasm in Kashmiris with the body and the antral regions as the commonest sites, nodular mass the commonest morphological type (as on endoscopy), adenocarcinoma the commonest histological type with no significant association between *H. pylori* infection and gastric cancer. At present, it is not possible to give an exact etiology of the high risk of gastric cancer in the valley. However, the peculiar geography and some special dietary habits with a possible familial predisposition may have a bearing on its etiology. But, further studies are needed to find correlation between the possible etiological factors and the occurence of this common neoplasm.

### *References*

[1] Mayor, R.J. Gastrointestinal tract cancer. In: *Harrison's Principles of Internal Medicine*. McgrawHill 14th edn., 1998: pp. 568–578.

[2] Devesa, S.S. and Silverman, D.T. Cancer incidence and mortality trends in the United States (1935–74). *Natl. Cancer Inst.* 1978; **60**: 545–571.

[3] Dunham, L.J. and Bailer, J.C., III World map of cancer mortality rates and frequency ratio. *J. Natl. Cancer Inst.* 1968; **41**: 155–203.

[4] Fuchs, C.S. and Mayor, R.J. Gastric carcinoma. *N. Engl. J. Med.* 1995; **333**: 32.

[5] Mohammad, A.E., Al-Karawi, A., Ahmad, A.M.M. *et al. Helicobacter pylori*: incidence and comparison of three diagnostic methods in 196 Saudi patients with dyspepsia. *Hepato-Gastroenterol.* 1994; **41**: 48–50.

[6] Khuroo, M.S., Zargar, S.A., Mahagen, R. *et al.* High incidence of oesophageal and gastric cancer in Kashmir in a population with special personal and dietary habits. *Gut* 1992; **33**: 11–15.

[7] Japan Research Society for gastric cancer, General rules for the gastric cancer study in surgery and pathology. *Jap. J. Surgery* 1981; **2**: 2.

[8] Malik, G.M., Kadla, S., Mubarik, M. *et al. Helicobacter pylori* infection in endoscopic biopsy specimens of gastric cancer: A preliminary evaluation in a high risk population of Kashmir Valley. *Diagnostic and Therapeutic Endoscopy* 1997; **4**(1): 35–42.

[9] Mitchell, H.M. The epidemiology of *H. pylori* infection and its relation to gastric cancer. In Goodin, C.S. and Wormslay, B.W. (Eds.) *H. pylori* – biopsy and clinical practices, Florida: CRC Press 1993; 95–114.

[10] Census of India 1981. Series 8, Jammu and Kashmir paper 1 of 1981 provisional population totals, pp. 1–41.

[11] Malhotra, S.L. Geographical distribution of gastrointestinal cancers in India with special reference to causation. *Gut* 1967; **8**: 361–372.

[12] Correa, P. Modulation of gastric carcinogenesis; updated model based on intragastric nitrosation. In: Bartsch, H., O'Neill, I. and Schulte-Hermann, R. (Eds.), *Relevance of N-nitroso Compounds to Human Cancer: Exposure and Mechenisms*, IARC scientific publication no. 84. Lyon: IARC, 1987; pp. 485–491.

[13] Ticiker, A.K., Siddiqi, M. and Preussmann, R. Occurence of volatile. N-nitrosoamines in dried chilies. *Cancer Lett.* 1988; **38**: 271–273.

[14] Siddiqi, M., Tricker, A.R., Kumar, R. *et al.* Dietary sources of N-nitrosoamines in a high risk area oesophageal cancer, Kashmir, India. In: O'Neill, I.K., Chen, J.S., Lu, S.H. and Bartsch, H. (Eds.) *Relevance to Human Cancer of N-nitroso Compounds, Tobacco Smoke and Mycotoxins*, IARC publication no. 105, Lyon: IARC, 1990; pp. 235–237.

[15] Siddiqi, M., Tricker, A.R. and Preussmann, R. Formation of N-nitroso compounds under stimulated gastric conditions from Kashmiri food stuffs. *Cancer Lett.* 1988; **39**: 359–365.

[16] Mirvish, S.S., Wallcave, L., Eagen, M. *et al.* Ascorbate nitrite reactions: A possible means of the formation of carcinogenic N-nitroso compounds. *Science* 1972; **177**: 65–68.

